# Role of Proteins in Oncology: Advances in Cancer Diagnosis, Prognosis, and Targeted Therapy—A Narrative Review

**DOI:** 10.3390/jcm13237131

**Published:** 2024-11-25

**Authors:** Magdalena Kędzierska, Magdalena Bańkosz

**Affiliations:** 1 Department of Chemotherapy, Medical University of Lodz, Copernicus Memorial Hospital of Lodz, 90-549 Lodz, Poland; 2 CUT Doctoral School, Faculty of Materials Engineering and Physics, Department of Material Engineering, Cracow University of Technology, 37 Jana Pawla II Av., 31-864 Krakow, Poland

**Keywords:** proteins in oncology, protein biomarkers, targeted therapy, monoclonal antibodies

## Abstract

Modern oncology increasingly relies on the role of proteins as key components in cancer diagnosis, prognosis, and targeted therapy. This review examines advancements in protein biomarkers across several cancer types, including breast cancer, lung cancer, ovarian cancer, and hepatocellular carcinoma. These biomarkers have proven critical for early detection, treatment response monitoring, and tailoring personalized therapeutic strategies. The article highlights the utility of targeted therapies, such as tyrosine kinase inhibitors and monoclonal antibodies, in improving treatment efficacy while minimizing systemic toxicity. Despite these advancements, challenges like tumor resistance, variability in protein expression, and diagnostic heterogeneity persist, complicating universal application. The review underscores future directions, including the integration of artificial intelligence, advanced protein analysis technologies, and the development of combination therapies to overcome these barriers and refine personalized cancer treatment.

## 1. Introduction

The role of proteins in cell biology and cancer mechanisms has become a key area of research in oncology, especially in the context of their application in diagnosis, prognosis, and targeted therapy [1]. Proteins are fundamental macromolecules with a wide range of biological functions, from structural to regulatory and enzymatic. Their role in maintaining cellular homeostasis means that any change in their structure, quantity, or function can lead to pathological consequences, including the formation of cancer [2,3]. In oncology, proteins not only play a role in cancer progression, but are also fundamental diagnostic biomarkers and therapeutic targets [4].

Proteins are key cancer biomarkers because their presence, structure, or expression can indicate the presence of disease or its specific characteristics, such as stage, aggressiveness, or ability to metastasize. Protein biomarkers are used to diagnose cancer at an early stage, monitor response to treatment, and assess prognosis. Key biomarker proteins include HER2 in breast cancer, PSA in prostate cancer, and CA-125 in ovarian cancer [5,6]. Their detection and analysis allow faster and more precise identification of cancer and assessment of its molecular characteristics. Recent advancements in protein analysis technologies, including mass spectrometry, immunological assays, and high-throughput proteomics, have significantly enhanced the ability to detect and study cancer-associated proteins with improved accuracy and efficiency. These technologies enable a deeper understanding of protein expression and post-translational modifications, facilitating the discovery of novel biomarkers and therapeutic targets [7,8,9]. The role of proteins in cancer diagnosis is particularly relevant in the context of the growing interest in liquid biopsy, which allows biomarker analysis without the need for invasive tissue biopsy [10,11].

Proteins play key roles in the mechanisms involved in neoplastic transformation; that is, the transition of a healthy cell into a cancerous cell. At the molecular level, these processes can be initiated by mutations in genes encoding proteins that regulate the cell cycle, apoptosis (programmed cell death), and the cellular stress response. Key mechanisms of tumorigenesis include dysfunction of proteins such as tyrosine kinases (e.g., EGFR, HER2) and transcription factors (e.g., p53) that regulate the transcription of genes responsible for cell proliferation, differentiation, and death. Mutations and overexpression of proteins such as EGFR (epidermal growth factor receptor) are associated with various types of cancers, including lung and colorectal cancers. Increased expression of EGFR may increase the ability of cancer cells to proliferate and escape immune control [12,13,14]. Another important example is the BCR-ABL oncoprotein, which arises from a chromosomal translocation and is crucial in the pathogenesis of chronic myeloid leukemia (CML) [15]. The protein has tyrosine kinase activity, which leads to uncontrolled cell growth. The case of BCR-ABL is also the first documented success of targeted therapy, where the tyrosine kinase inhibitor imatinib proved effective in inhibiting its activity, ushering in a new era of precision therapies in oncology [16].

The ability of tumors to evade recognition by the immune system is one of the key mechanisms that enable them to survive and thrive. In this context, proteins play a fundamental role, as many of them have a function in modulating the immune response [17,18]. Proteins such as PD-L1 and CTLA-4 are critical to the immunosuppressive mechanisms of cancer, which allow cancer cells to escape the body’s immune response. PD-L1, a protein detected on the surface of some cancer cells, binds to the PD-1 receptor on T cells, leading to their inactivation. This mechanism has been widely used in immuno-oncology therapy, in which checkpoint inhibitors such as PD-1 and PD-L1-blocking antibodies restore the immune system’s ability to attack cancer cells [19,20,21].

The use of proteins as therapeutic targets in oncology represents a breakthrough advance in cancer treatment. Targeted therapies, which are based on the specific molecular characteristics of tumors, allow for a more effective and precise treatment approach compared to classical therapies such as chemotherapy or radiotherapy [21,22]. With a better understanding of protein signaling pathways, such as tyrosine kinases and growth factor receptors, targeted drugs have been developed that specifically act on tumor proteins. Imatinib (a BCR-ABL tyrosine kinase inhibitor), trastuzumab (a monoclonal antibody directed against HER2), or PD-1/PD-L1 inhibitors are examples of therapies that have revolutionized the treatment of patients with various types of cancers [23]. Targeted therapy minimizes side effects because it mainly affects proteins present or overexpressed in cancer cells, rather than in healthy tissues [13,24].

In addition, developments in molecular technologies, such as next-generation DNA sequencing (NGS) and proteomic analysis, are making it possible to more precisely characterize the protein profile of each patient’s tumors [25]. As a result, treatment personalization approaches that take into account an individual’s molecular profile are becoming increasingly important. Personalization of therapy allows drugs and therapeutic strategies to be tailored to the specific mutations and protein abnormalities present in a patient’s tumor cells, thereby increasing treatment efficacy and reducing side effects [16,26].

As biomolecular technologies advance, the potential for discovering new biomarkers and protein-based therapeutic targets is growing. Current research is focused on identifying proteins responsible for mechanisms of treatment resistance and those that may play a role in cancer progression and metastasis formation. Protein analysis techniques such as proteomics and mass spectrometry, as well as modern approaches to protein modification (e.g., CRISPR-Cas9 in gene editing), are opening up new research perspectives.

**Figure 1:**
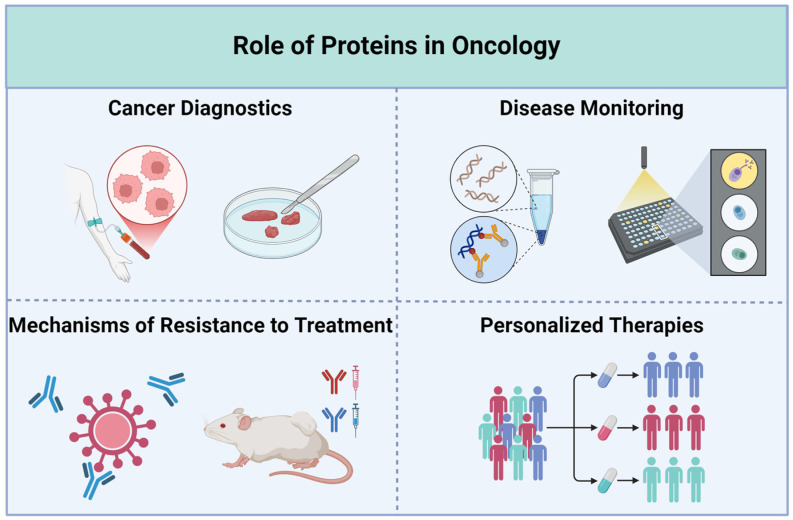
Schematic of protein use in oncology.

Modern molecular oncology is focused on studying the entire spectrum of cancer proteins, which may allow a better understanding of cancer biology and the development of more effective treatment strategies. In summary, proteins play a key role in oncology, affecting every step of diagnosis, prognosis, and treatment (Figure 1). A review of current research on proteins in the context of cancer can help us understand their importance and application potential. This review delves into the pivotal role of proteins in cancer biology, emphasizing their applications in diagnosis, prognosis, and targeted therapies. By exploring key examples such as HER2, PSA, and CA-125, it illustrates how protein biomarkers have revolutionized early detection, treatment monitoring, and personalized medicine. These biomarkers were specifically chosen for their established clinical relevance and significant impact on common cancers such as breast, prostate, and ovarian cancers, where they have transformed personalized treatment strategies. The article further discusses advancements in technologies like mass spectrometry and liquid biopsy that have enhanced the precision of protein analysis. It also addresses challenges such as tumor resistance and variability in protein expression, while outlining emerging strategies, including combination therapies and AI-driven approaches, to improve personalized cancer care. This targeted focus ensures a thorough exploration of the most impactful biomarkers and strategies, providing insights into current challenges and future directions in oncology.

## 2. Importance of Proteins in Cancer Diagnostics

Cancer diagnosis plays a key role in the successful treatment and management of the disease, and proteins are one of the most important components of this diagnosis. Detecting specific proteins that are characteristic of cancer cells or that appear as a result of cancerous changes in the body makes it possible to identify the disease at an early stage, assess the aggressiveness of the tumor, monitor the progress of therapy, and predict the prognosis.

### 2.1. Examples of Protein Biomarkers

Protein biomarkers, thanks to their diversity and specificity, are the foundation of modern diagnostic methods in oncology. The use of advanced technologies makes it possible to precisely detect and analyze the expression and modification of proteins, which is crucial for individualizing the diagnosis and treatment of cancer patients [27]. Protein biomarkers are proteins whose presence, quantity, or structure indicates the presence of disease, tumor stage, or response to treatment. Proteins such as PSA (prostate-specific antigen) in prostate cancer, HER2 in breast cancer, CA-125 in ovarian cancer, and AFP (alpha-fetoprotein) in liver cancer are particularly important in cancer diagnosis [28,29].

PSA is a glycoprotein produced by epithelial cells of the prostate gland, whose function is to liquefy semen, facilitating sperm motility [30,31]. Under physiological conditions, PSA concentration in the blood is relatively low; however, in pathological states such as prostate cancer, benign prostatic hyperplasia (BPH), or prostatitis, PSA levels increase significantly [32]. PSA is one of the most widely used biomarkers in oncology, especially in screening men over the age of 50. The PSA test is widely used not only for diagnostic purposes but also for monitoring tumor progression and evaluating treatment effectiveness. In cases of tumor recurrence or treatment failure, PSA levels rise, enabling early detection of disease relapse [33,34]. However, elevated PSA levels are not entirely specific to prostate cancer, so additional markers, such as the free-to-total PSA ratio (fPSA/tPSA) and PSA velocity (PSA increase rate over time), are increasingly used in diagnostics [35]. It is worth noting that the free-to-total PSA ratio (F/T PSA) and the percentage of free PSA (%fPSA) can serve as useful auxiliary indicators in diagnostics; however, they do not provide a sufficient basis for independently diagnosing prostate cancer. Phiri-Ramongane and Khine demonstrated that an F/T PSA cutoff of 25% has limited diagnostic value, resulting in a high number of unnecessary biopsies for patients with PSA levels in the so-called “gray zone” (4–10 ng/mL). The authors recommend that biopsy decisions be based on a broader clinical assessment rather than solely on PSA levels [36]. The study by Prcic et al. indicated that lowering the F/T PSA threshold to ≤16% could increase diagnostic specificity while maintaining acceptable sensitivity. They reported that, at this level, sensitivity reached 72.3% and specificity was 50.4%, which suggests some diagnostic value for F/T PSA in distinguishing prostate cancer, although it remains insufficiently precise as a standalone indicator [37]. Conversely, the study by Omar et al. suggests that a %fPSA cutoff of 25% falls short of expectations for sensitivity and specificity in prostate cancer diagnostics, as fPSA levels may be similar in both benign and malignant cases, which limits its utility [38]. In summary, although F/T PSA and %fPSA are valuable supplementary indicators, their diagnostic effectiveness is limited, and their use should be complemented by other methods such as prostate palpation and biopsy. Diagnostic decisions based solely on these PSA indicators may lead to excessive interventions; therefore, an optimal approach should consider a broader clinical context to minimize the risk of unnecessary biopsies and improve the accuracy of prostate cancer diagnosis. Results reported in the literature suggest that the sensitivity and specificity of PSA as a biomarker for prostate cancer diagnosis can vary considerably depending on the diagnostic threshold used and additional parameters. For example, for a threshold value of 4.0 ng/mL, sensitivity ranged from 21% to 35%, while specificity for distinguishing benign prostatic hyperplasia from prostate cancer ranged from 20% to 25% at lower thresholds such as 2.5 ng/mL [39]. In contrast, PSA velocity (PSA velocity) analysis, where changes of 0.75 ng/mL per year were analyzed, showed a specificity of about 30% in detecting clinically significant cancer [31]. In contrast, in PSA-derived studies, the percentage of free PSA (%fPSA) reached a sensitivity of 95% with a threshold of 25%, but at the expense of a low specificity of 20%. These results demonstrate the potential of PSA and its derivatives in diagnosis, while highlighting the variability of these parameters depending on the methodology and criteria used [40].

Furthermore, PSA is used for prostate cancer screening, but it often yields false-positive results, which is why studies are being conducted to modify its application. The focus of Tosoian et al.’s study was on the use of the MyProstateScore 2.0 (MPS2) diagnostic test, which combines traditional PSA testing with the analysis of genes specific to aggressive prostate cancer, aimed at improving the diagnostic accuracy of prostate cancer and reducing the excessive number of biopsies. The study results demonstrated that MPS2 can reduce the number of unnecessary biopsies by 35–42% among patients with a low risk of aggressive disease, while also showing higher sensitivity in detecting high-grade cancers. The implementation of MPS2 allows for a more precise identification of patients requiring more intensive diagnostic procedures, minimizing the risk of overdiagnosis and optimizing the management of prostate cancer patient care [41]. Subsequently, Yoneyama et al. presented a study on the application of a specifically glycosylated form of PSA, designated as S23PSA (α2,3-sialylated PSA), as a biomarker for detecting clinically significant prostate cancer in men with elevated PSA levels. The study aimed to assess the diagnostic efficacy of S23PSA density (S23PSAD) and to compare it with traditional indicators, such as total PSA (tPSA) and PSA density (PSAD), as well as with MRI results in evaluating clinically significant cases of prostate cancer. The study results indicated that S23PSAD diagnostically outperformed tPSA and PSAD in identifying patients with aggressive forms of prostate cancer, achieving a higher area under the ROC curve (AUC), which reflects its improved sensitivity and specificity. In both retrospective and prospective studies, S23PSAD also enabled a reduction of unnecessary biopsies by approximately 13% when used in conjunction with MRI assessment (PI-RADS), suggesting that S23PSAD could be an effective addition to standard diagnostics, enhancing the selection of patients who truly require a biopsy [42].

HER2, or Human Epidermal Growth Factor Receptor 2, is a membrane receptor belonging to the epidermal growth factor receptor (EGFR) family, which regulates cell growth, differentiation, and survival. Overexpression or amplification of the HER2 gene is characteristic of aggressive forms of breast cancer and occurs in approximately 15–20% of cases. The increased amount of HER2 receptors on the surface of cancer cells leads to excessive activation of signaling pathways, resulting in rapid proliferation and the evasion of control mechanisms such as apoptosis [43]. The HER2 biomarker, a tyrosine kinase receptor, plays a significant role in the development and progression of various cancers, serving as a crucial target in targeted therapies. Numerous studies have investigated the significance of HER2 amplification and overexpression across different cancer types, from lung cancer to rare cases such as Paget’s disease of the skin, as well as the mechanisms of action and resistance to HER2-targeted drugs like trastuzumab [44,45]. The primary advantage of HER2 as a biomarker lies in its ability to identify patients who can benefit from targeted therapies such as trastuzumab, which significantly improves survival and treatment outcomes. However, its limitations include expression heterogeneity within tumors and the potential for resistance to HER2-targeted therapies, necessitating the development of combination treatments and comprehensive biomarker monitoring [13,46].

Li et al. analyzed HER2 amplification and mutations as therapeutic targets in lung cancer, finding that HER2 amplification and mutations are independent molecular alterations that rarely co-occur. They concluded that patients with HER2 mutations in lung cancer may be better suited for targeted therapy than those with amplification alone, emphasizing the importance of precise patient selection in effective targeted treatment [47]. Valabrega et al. focused on trastuzumab’s mechanism of action in HER2-positive breast cancer, analyzing both the drug’s effects and the development of resistance. The study suggests that trastuzumab works by inhibiting HER2-dependent signaling pathways and inducing immune-mediated cytotoxicity, but resistance may emerge through alternative pathway activation or loss of PTEN activity. These findings highlight the importance of biomarker monitoring and considering combination therapy to overcome resistance [48]. Tanaka et al. studied HER2 overexpression in the rare skin condition extramammary Paget’s disease (EMPD), demonstrating that HER2 is overexpressed in aggressive EMPD cases with lymph node metastasis. Their findings suggest that HER2 could serve as a valuable prognostic biomarker in EMPD and indicate the potential efficacy of HER2-targeted therapies in cases with high biological aggressiveness [49]. Lastly, Lee et al. examined HER2 status in gastric cancer, focusing on expression heterogeneity across biopsy and resection samples. They found that HER2 overexpression is more common in intestinal-type gastric cancers and tumors located near the gastroesophageal junction. The results underscore the need for thorough HER2 evaluation using multiple samples due to this biomarker’s heterogeneity within tumor tissue [50]. These studies demonstrate HER2’s diverse applications and limitations as a biomarker in various cancers, highlighting the importance of tissue and molecular contexts in achieving effective targeted treatment.

CA-125 (Cancer Antigen 125) is a high-molecular-weight glycoprotein, primarily expressed in the epithelial cells of the peritoneum, pleura, and pericardium. It is widely used as a biomarker in ovarian cancer, particularly in the diagnosis and monitoring of patients with advanced stages of the disease [51,52]. CA-125 is not specific to ovarian cancer; elevated levels can also be detected in other pathological conditions, such as endometritis, endometriosis, and certain other cancer types (e.g., pancreatic and lung cancers). Despite limitations in specificity, CA-125 remains a crucial tool for monitoring treatment response and detecting ovarian cancer recurrence [53]. Its relatively short half-life allows for real-time tracking of level changes, making it useful for evaluating the effectiveness of postoperative and chemotherapy treatments. Research on CA-125 continues, aiming to enhance its diagnostic accuracy by combining it with other markers or technologies, such as liquid biopsy [54,55].

**Figure 2:**
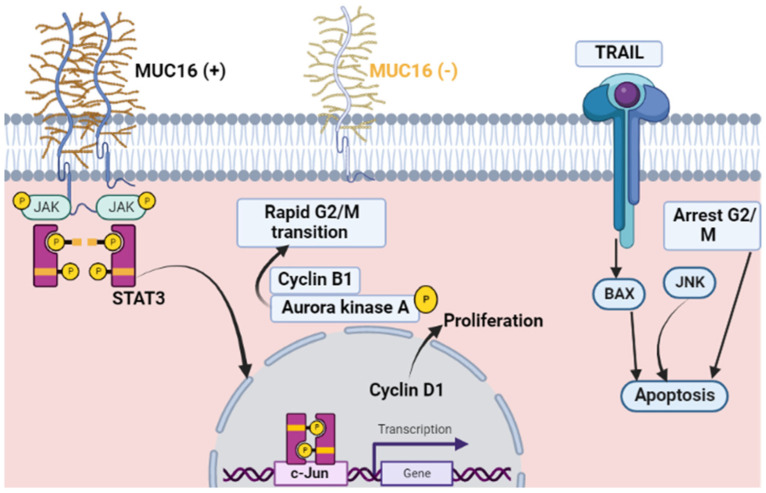
MUC16 expression in cancer [56].

MUC16, of which the biomarker CA-125 is a fragment, plays a significant role in cancer progression by stimulating cell division and regulating key signaling pathways. MUC16 expression supports the proliferation of cancer cells through binding with the non-receptor tyrosine kinase JAK2, which leads to the phosphorylation of the transcription factor STAT3 and activation of c-Jun, responsible for the expression of Cyclin D1. MUC16 also influences the G2/M transition in the cell cycle by regulating Cyclin B1 levels and the activity of Aurora kinase A. Reduced expression of MUC16 can lead to the accumulation of malignant cells in the G2/M phase, resulting in apoptosis through the JNK signaling pathway. Figure 2 illustrates these mechanisms of MUC16 activity [56]. The main advantage of CA-125 lies in its ability to track disease progression and recurrence with a relatively straightforward and non-invasive test. However, its specificity is limited, as elevated levels can occur in benign gynecological conditions, necessitating its use alongside additional diagnostic markers to improve accuracy [57,58].

The tumor marker CA-125 is one of the most commonly used biomarkers in the diagnosis of ovarian cancer, but its sensitivity and specificity are variable depending on the diagnostic threshold used and the stage of the disease. For example, CA-125 has achieved a sensitivity of 88% and specificity of 57% in detecting suspicious cystic masses in the ovary [59]. In contrast, in more advanced analyses, CA-125 showed a sensitivity of 95.3% and specificity of 99.4% in the diagnosis of ovarian cancer, highlighting its high clinical utility [60]. For early detection of serous neoplasms, the marker reached a sensitivity and specificity of 94% each [61]. Nevertheless, its effectiveness decreases in the early stages of cancer, where the sensitivity is about 70% [62]. In order to improve specificity, CA-125 can increasingly be combined with other biomarkers to more accurately differentiate between benign and malignant lesions [63]. These findings suggest that CA-125 should be used in combination with additional markers to achieve optimal diagnostic performance.

AFP (Alpha-Fetoprotein) is a fetal protein produced in the liver and yolk sac during embryonic development. Under normal conditions, AFP levels in adults are low; however, they increase significantly in cases of primary liver cancer (hepatocellular carcinoma, HCC) and germ cell tumors of the testes and ovaries [64]. AFP is particularly useful as a biomarker for diagnosing HCC, especially in countries with a high prevalence of cirrhosis and hepatitis B and C, which are risk factors for this cancer. AFP also has a prognostic function and is used to monitor treatment response and detect early cancer recurrence [65,66]. Elevated AFP levels can also occur in non-cancerous conditions, which limits its specificity; however, its combination with other markers, such as DCP (des-gamma-carboxyprothrombin), enhances diagnostic accuracy for HCC [67]. The primary advantage of AFP is its ability to identify high-risk patients and facilitate early detection of HCC, especially in populations with pre-existing liver diseases. However, its specificity is limited, as elevated AFP levels are also observed in non-cancerous liver conditions, necessitating its use in combination with other biomarkers like DCP for improved diagnostic precision [68,69].

Liu et al. focused on using AFP as a target for CAR T cell therapy in liver cancer treatment. Because AFP is primarily an intracellular protein, it is challenging to target directly with conventional antibody therapies. To overcome this limitation, the research team developed a specific antibody directed at the AFP-MHC class I complex and implemented it in the form of CAR T cells. The results showed that AFP-CAR T effectively eliminated AFP-expressing cancer cells in mouse models, highlighting the potential of this strategy for treating solid tumors that are resistant to traditional therapies [70]. In another study, Chen et al. examined the role of AFP in AFP-positive gastric cancer (AFPGC), a rare yet highly aggressive subtype of this cancer. The study found that high AFP levels correlate with the expression of ANGPTL6, a protein that promotes angiogenesis and tumor progression by activating the ERK1/2 and AKT signaling pathways. These mechanisms support cancer cell migration and blood vessel development within the tumor, making ANGPTL6 a potential therapeutic target in AFPGC and reinforcing AFP’s value as a prognostic biomarker in this rare malignancy [71]. Finally, Sun et al. conducted a study on the AFP ratio as a prognostic indicator of survival in HCC patients following surgical resection. The AFP ratio, which measures the proportion of preoperative to postoperative AFP levels, proved to be a strong predictor of disease-free survival (DFS) and overall survival (OS). The study showed that a higher AFP ratio is associated with poorer outcomes, suggesting AFP’s utility not only in diagnostics but also in monitoring post-surgical treatment response and predicting disease progression [72].

CEA (Carcinoembryonic Antigen) is a high-molecular-weight surface glycoprotein normally present in fetal tissues. In adults, CEA shows low expression in most healthy tissues; however, its levels increase in many types of cancers, including colorectal, lung, pancreatic, gastric, and breast cancers [73]. Specifically, CEA plays a key role in the diagnosis of colorectal cancer and is widely used to monitor postoperative recurrence and assess the effectiveness of chemotherapy [74]. CEA is primarily used as a monitoring marker, as its levels correlate with disease progression and the presence of metastases. Although CEA is not specific to any particular type of cancer, combining it with other markers (e.g., CA19-9 in pancreatic cancer) allows for a more precise assessment of the patient’s condition [75]. Currently, research is underway to develop more sensitive CEA tests that could detect even small changes in CEA levels in real time [76]. Moreover, CEA is valuable for tracking disease progression and treatment response in colorectal cancer, but its limited specificity necessitates combining it with other biomarkers for improved accuracy [77,78].

In a study conducted by Van der Willik et al., the relationship between CEA levels and the risk of dementia was analyzed using a cohort of 6692 participants from the Rotterdam Study who were cancer-free. The results indicated that elevated CEA levels were correlated with an increased risk of dementia, which may suggest a biological link between early-stage cancer and the development of dementia. The researchers concluded that higher CEA levels might indicate the presence of early, undiagnosed cancers in patients, potentially explaining a positive association between cancer and dementia, contrasting with previous hypotheses about their negative correlation [79]. Meanwhile, the study by Jozghorbani et al. focused on developing an innovative electrochemical immunosensor based on graphene oxide, enabling the detection of CEA in clinical samples. This sensor utilizes electrochemical impedance spectroscopy (EIS) and cyclic voltammetry (CV) for detection, achieving high sensitivity in the range of 0.1 to 5 ng/mL and a detection limit of 0.05 ng/mL. The study results showed that the graphene-based sensor is effective in identifying CEA in serum samples, making it a potentially fast and cost-effective diagnostic tool for monitoring CEA levels, particularly useful in cancers such as colorectal cancer. This method could simplify and improve the early diagnosis and monitoring of cancer progression [80]. In the study conducted by Osumi et al., the relationship between circulating tumor DNA (ctDNA) levels and carcinoembryonic antigen (CEA) levels was analyzed in patients with metastatic colorectal cancer (mCRC). The study aimed to determine the clinical value and differences between ctDNA and CEA by assessing the correlation between ctDNA and CEA levels and their association with tumor size. The study included 110 patients with mCRC who were undergoing chemotherapy. Results showed that the correlation coefficient between ctDNA and CEA was 0.53, indicating a moderate concordance between these markers. However, this correlation was lower in patients without liver or lymph node metastases, suggesting that the presence of liver metastases influences the levels of these markers. It was concluded that although both markers are useful in monitoring mCRC patients, their diagnostic value varies depending on the location of metastases and tumor volume [81].

In addition, it is also worth mentioning that tumor-specific markers such as JAK2, CALR, and MPL mutations are being widely studied. These markets provide greater diagnostic precision in chronic myeloproliferative neoplasms (cMPN). These mutations not only underlie the pathogenesis of these hematologic cancers, but also serve as reliable biomarkers for diagnosis, prognosis, and targeted therapeutic strategies. JAK2 V617F, the most prevalent mutation in cMPNs, is detected in over 95% of polycythemia vera (PV) cases and approximately 50–60% of essential thrombocythemia (ET) and primary myelofibrosis (PMF) cases. This gain-of-function mutation results in the constitutive activation of the JAK-STAT signaling pathway, promoting uncontrolled proliferation of myeloid cells. Beyond its diagnostic significance, the presence of the JAK2 mutation also carries prognostic implications, as it is associated with an increased risk of thrombotic events and disease progression [82,83]. In cases negative for JAK2 mutations, alterations in CALR (calreticulin) and MPL (myeloproliferative leukemia protein) genes serve as alternative diagnostic markers. CALR mutations, found in approximately 20–25% of ET and PMF cases, predominantly affect exon 9 of the gene, leading to frameshift mutations [84,85]. These alterations result in a mutant protein with oncogenic properties, particularly through aberrant activation of the JAK-STAT pathway. CALR mutations have been associated with a more favorable prognosis in comparison to JAK2-positive cases, especially with respect to thrombotic complications. Mutations in the MPL gene, which encodes the thrombopoietin receptor, occur in 3–8% of ET and PMF cases. These mutations, most notably W515L/K, similarly result in the constitutive activation of downstream signaling pathways, driving uncontrolled megakaryocyte and platelet proliferation. The diagnostic utility of MPL mutations is particularly relevant in cases lacking both JAK2 and CALR mutations. The integration of these tumor-specific biomarkers has significantly enhanced the diagnostic framework for cMPNs, allowing for more precise classification and tailored therapeutic approaches. For instance, targeted inhibitors of JAK2, such as ruxolitinib, have emerged as cornerstone treatments in JAK2-positive cMPNs, providing symptomatic relief and reducing splenomegaly. The molecular mutations of CALR and MPL are also being explored for novel therapeutic targets, with ongoing research aiming to develop treatments that directly inhibit their pathogenic effects [86,87].

### 2.2. Advantages and Limitations of Biomarkers

Protein biomarkers play a crucial role in modern oncology diagnostics, offering significant advantages that make them invaluable tools for cancer detection and monitoring. One of the most important benefits of protein biomarkers is their ability to detect cancers at an early stage, often before visible clinical symptoms appear. For example, prostate-specific antigen (PSA), when present in elevated concentrations in the blood, may indicate early-stage prostate cancer. Early detection allows for prompt medical intervention, which in many cases significantly increases the chances of effective treatment and extended patient survival. Biomarkers such as CA-125, used in ovarian cancer, and AFP, used in liver cancer diagnosis, enable the identification of cancers even in high-risk groups where rapid diagnosis is essential [88]. Another advantage of protein biomarkers is their ability to monitor disease progression and therapy effectiveness. This allows for a precise assessment of whether the applied treatment is achieving the intended effects and enables a quick response in cases of poor response or recurrence. For instance, carcinoembryonic antigen (CEA) is used to monitor health status changes in colorectal cancer patients. A decrease in biomarker levels after therapy can indicate treatment success, whereas an increase may suggest a possible recurrence, allowing for early intervention. Regular monitoring of biomarkers provides a dynamic evaluation of treatment effectiveness, giving clinicians better control over the course of therapy. An essential benefit of protein biomarkers is their minimal invasiveness [89,90]. Many biomarkers can be detected in bodily fluids such as blood serum, urine, or cerebrospinal fluid, eliminating the need for tissue sampling. This technique, especially evident in liquid biopsy, allows the analysis of circulating proteins or tumor DNA, enabling precise and frequent patient monitoring in a less burdensome manner. Liquid biopsy is particularly valuable when traditional tissue biopsy is challenging or risky for the patient. Finally, protein biomarkers play a key role in the personalization of cancer treatment, allowing therapy to be tailored to the specific molecular characteristics of a patient’s tumor. For instance, the presence of the HER2 receptor in breast cancer enables the use of trastuzumab, a targeted drug that specifically acts on cells with overexpression of this protein, resulting in greater treatment effectiveness compared to non-selective therapies [91]. Similarly, the expression of PD-L1 protein in cancers, such as lung cancer, qualifies patients for immunotherapy using immune checkpoint inhibitors, enhancing treatment effectiveness. By personalizing treatment based on biomarker profiles, oncology medicine is becoming increasingly precise and effective, allowing patients to achieve better therapeutic outcomes with minimal side effects. Through their broad applications, protein biomarkers contribute not only to improved diagnostic outcomes but also to the optimization and personalization of therapies, thereby increasing the chances of success in cancer treatment [92,93].

Although protein biomarkers are an essential diagnostic and therapeutic tool in oncology, their application comes with significant limitations that affect their effectiveness and precision. One of the main challenges is their low specificity. Many protein biomarkers can show elevated levels not only in the presence of cancer but also in non-cancerous conditions such as infections, inflammation, or benign changes. For instance, prostate-specific antigen (PSA), commonly used for prostate cancer detection, may present elevated levels due to benign prostatic hyperplasia or prostatitis, leading to false-positive results that can unnecessarily increase patient stress and lead to additional invasive testing. Another issue is the considerable variability in biomarker levels among patients, which can lead to false-negative diagnostic results. The levels of markers such as CA-125 in ovarian cancer may vary between patients depending on individual biological characteristics and disease progression. In some cases, a biomarker may not indicate the presence of cancer even if it exists, limiting its use as the sole indicator in diagnosis or disease monitoring. This variability also complicates the establishment of clear diagnostic and prognostic criteria, impacting result standardization and the ability to compare outcomes [94,95]. Recent advances, however, address these limitations. Combining multiple biomarkers has shown promise in improving diagnostic specificity. For example, using panels of markers such as HE4 with CA-125 improves the accuracy of ovarian cancer diagnosis, reducing false-positive rates by differentiating malignant from benign conditions more effectively [96,97]. Liquid biopsy is another significant advancement, enabling the detection of circulating tumor DNA (ctDNA) and proteins. This technique provides a more comprehensive molecular profile of the tumor, allowing for more accurate monitoring of disease progression and therapeutic response [98,99]. A further limitation is the lack of standardization in analytical methods for protein biomarker measurement, leading to discrepancies in results between laboratories. Despite technological advances, analytical procedures are not always standardized, making it difficult to compare results from different centers and monitor patients over time. Standardization is especially crucial when protein biomarkers are used to monitor treatment, such as assessing therapy effectiveness using markers like CEA or PSA. Lack of standardization can result in incorrect conclusions about cancer progression or regression, influencing therapeutic decision-making [100,101]. The final concern is the high cost and limited availability of certain protein biomarkers, especially those requiring advanced technologies like mass spectrometry. High laboratory testing costs restrict accessibility, particularly in facilities with limited budgets or in developing countries. For example, costly tests for biomarkers such as HER2 in breast cancer may not be available to all patients, limiting the possibilities for targeted therapy. Limited accessibility to these tests can hinder treatment personalization and access to modern diagnostic methods for patients, reducing the potential benefits of optimized therapy matching. The development of cost-effective and widely accessible methods, such as simplified ctDNA detection kits, is expanding biomarker availability, enabling broader application in diverse healthcare settings [102,103]. The disadvantages of protein biomarkers, such as low specificity, patient-to-patient variability, lack of standardization, high costs, and limited availability, pose significant challenges to their use. These issues require further research, technological advancements, and the development of standards for their evaluation to fully leverage their potential in cancer diagnosis and treatment [104,105]. Moreover, the variability of CA-125 biomarker levels among patients represents a significant limitation in its application as an indicator for monitoring ovarian cancer. Despite its widespread use to assess therapeutic response and disease progression, its clinical interpretation is fraught with challenges stemming from biological and analytical variability. Studies have shown that CA-125 levels are influenced by both biological factors and laboratory precision. Analytical variability increases proportionally with absolute CA-125 levels, but relative variability, expressed as the coefficient of variation, remains relatively constant at 1.5–2.5%, regardless of the marker’s range [106]. Moreover, the relationship between CA-125 levels and clinical symptoms is inconsistent. In numerous cases, a significant rise in CA-125 levels, even by several magnitudes, did not lead to the appearance of clinical symptoms, while in other situations, patients remained asymptomatic for extended periods despite persistently elevated marker levels [107]. Such biological variability significantly complicates the prediction of short-term disease progression based on a single measurement of CA-125 or even its trend over time. Furthermore, an increase in CA-125 levels, especially within borderline or slightly elevated ranges, is insufficient to conclusively infer disease progression [106,108]. Combining CA-125 with emerging biomarkers or advanced imaging techniques is currently being investigated to improve its diagnostic and prognostic value, offering a more holistic view of tumor dynamics [109,110]. A summary of the advantages and disadvantages of selected protein biomarkers is presented in Table 1.

## 3. Role of Proteins in Cancer Prognosis

Proteins play a fundamental role in cancer prognosis, providing essential information about disease progression, risk of progression, and treatment response. Through their involvement in biological processes and cell signaling, proteins can serve as prognostic biomarkers, enabling the assessment of prognosis and the adjustment of therapeutic strategies for individual patients. Prognostic proteins, such as p53, Ki-67, and PD-L1, are crucial in the personalization of oncology therapies and contribute to more effective disease management.

### 3.1. Examples of Prognostic Proteins

Prognostic biomarkers are proteins whose presence, overexpression, mutations, or modifications impact the assessment of disease progression and prognosis for cancer patients. Prognostic proteins can indicate tumor aggressiveness, growth rate, and metastatic potential. Their use enables the identification of high-risk patients who require more intensive therapy, as well as patients with less aggressive cancers, for whom less invasive therapeutic approaches may be applied. In the context of various cancer types, prognostic proteins play a crucial role in assessing individual risk of disease progression. For instance, in breast cancer, HER2 receptor overexpression correlates with greater tumor aggressiveness and is associated with a poorer prognosis. Conversely, in colorectal cancer, high levels of carcinoembryonic antigen (CEA) can indicate an increased risk of metastasis and recurrence following surgical treatment. Understanding the expression and role of these proteins in cancers allows physicians to plan therapies more precisely and closely monitor high-risk patients [111,112].

Protein p53 is a protein of crucial importance in cell cycle control and the response to DNA damage. Mutations in the gene encoding p53 are among the most common mutations in cancers and lead to cancer cells evading mechanisms that regulate cell division, promoting uncontrolled tumor growth [113]. The presence of p53 mutations is a prognostic factor in many types of cancer, including lung, colorectal, and pancreatic cancers, and is associated with poorer outcomes. The prognostic implications of p53 mutations are significant, as their presence often correlates with resistance to conventional chemotherapy, necessitating the use of alternative therapeutic strategies [114,115].

**Figure 3:**
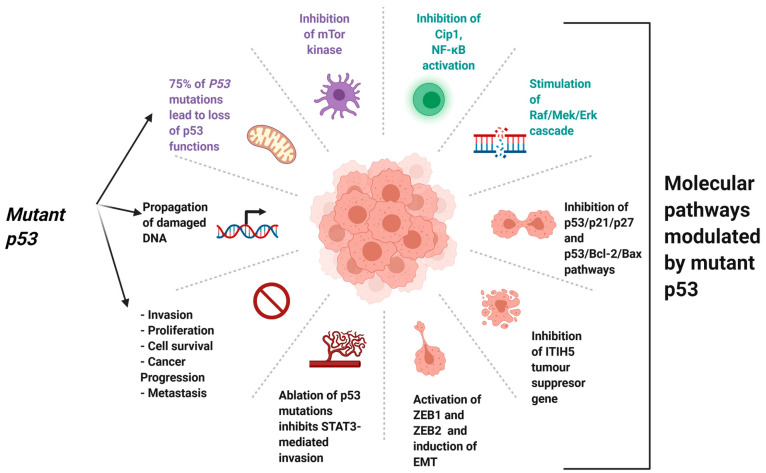
Impact of various p53 mutations on its function and cancer progression [116].

Figure 3 illustrates various mutations of the p53 gene and their effects on its function and oncogenic activity, highlighting how TP53 gene mutations, which deactivate the wild-type p53 gene in 75% of cases, promote invasion, proliferation, survival, cancer progression, and metastasis by interacting with key molecular pathways such as mTOR inhibition, NF-κB activation, and induction of epithelial–mesenchymal transition [116].

The study conducted by Ben Rejeb et al. examined the role of the p53 protein as a prognostic marker in prostate cancer, focusing on p53 expression using immunohistochemical methods. The results showed that abnormal p53 expression, manifested as either overexpression or lack of expression, was correlated with poorer patient prognosis and resistance to androgen deprivation therapy, which is commonly used in prostate cancer treatment. It was found that patients with aberrant p53 expression had a higher risk of aggressive disease progression, potentially leading to a faster progression and a higher likelihood of recurrence after treatment. This study suggests that p53 expression may play a significant role in identifying high-risk patients with aggressive prostate cancer who may require more intensive treatment or frequent monitoring. In terms of prognosis, p53 may therefore serve as a valuable indicator for assessing progression risk and treatment response [117]. Next, Kim et al. analyzed the expression of p53 protein and TP53 mutations in the context of colorectal cancer (CRC), using both immunohistochemistry and next-generation sequencing (NGS) to identify TP53 mutations. The results showed that p53 protein expression was a stronger predictor of patient survival than the mere presence of TP53 mutations, suggesting that p53 expression levels can provide more precise prognostic information than TP53 gene mutations alone. They found that high levels of p53 expression were associated with poorer patient survival, indicating a more aggressive course of the disease. p53 expression in colorectal cancer may thus serve as a prognostic tool, aiding in the identification of patients at a higher risk of adverse disease progression, allowing for more accurate treatment planning and potentially earlier application of more aggressive interventions [118]. Schaafsma et al. developed the p53 Deficiency Score (PDS), which is based on the expression of genes regulated by p53, as an alternative approach to prognosis assessment in various cancers. The researchers found that PDS is a more effective predictor of clinical outcomes than TP53 mutation status, especially in breast, liver, lung, and cervical cancers. The results indicated that PDS better correlates with disease progression risk and treatment response. The p53 Deficiency Score indirectly measures p53 functionality based on the expression of genes under its regulation, allowing a more accurate assessment of the biological activity of this protein in cancer cells. The prognostic value of PDS lies in its ability to predict whether the cancer is more prone to progression or if the patient is likely to have a favorable prognosis. This study highlights that a deficiency in p53 function, measured through its regulated genes, may have greater prognostic significance than simply the presence of mutations in the TP53 gene, suggesting the potential superiority of this method in the prognosis of various cancers [119]. Furthermore, intensive research is being conducted on the P53 Deficiency Score (PDS) to better understand the role of functional p53 deficiency in cancer development and its potential use as a prognostic and predictive biomarker. These studies primarily aim to determine the extent to which PDS can predict disease recurrence risk, cancer aggressiveness, and response to anti-cancer therapies. For instance, in studies on lung cancer (adenocarcinoma), PDS has demonstrated significant predictive capabilities, particularly in early-stage patients, enabling patient stratification by recurrence risk and facilitating the planning of more tailored therapeutic strategies [120,121]. Additionally, research on PDS in other cancer types, such as breast and colorectal cancers, indicates that functional p53 deficiency plays a critical role in the development of treatment resistance, including resistance to cisplatin-based chemotherapy. Specifically, studies are examining how PDS can help identify patients who may benefit from more intensive or targeted molecular therapies [122].

In the study conducted by Rojas et al., the impact of p53 protein isoforms on the prognosis of patients with newly diagnosed multiple myeloma was examined. The authors identified various p53 isoforms and investigated their relationship with patient survival. The results indicated that high expression of the shorter p53 isoforms was associated with better survival rates and a lower risk of disease progression. In contrast, high expression of TAp53β/γ isoforms was linked to shorter survival times and a higher risk of progression. These findings suggest that specific p53 isoforms may play a significant role in cancer prognosis, and that expression levels of certain isoforms can provide precise prognostic information for patients at high cytogenetic risk. These conclusions support the hypothesis that detailed analysis of p53 isoforms could add substantial prognostic value in assessing the risk of cancer progression [123].

Ki-67 is a proliferation marker that plays a significant role in assessing the divisional activity of cancer cells [124,125]. Its expression correlates with tumor growth rate; therefore, a high level of Ki-67 is prognostically unfavorable and indicates an aggressive course of the disease [126]. Ki-67 is widely used in evaluating prognosis for breast cancer patients, but its role in assessing proliferation also applies to other types of cancers, such as gliomas and neuroendocrine tumors. High Ki-67 levels can influence therapeutic decisions, as they indicate a higher risk of recurrence and metastasis [127].

In the study by Mighri et al., the role of Ki-67 in predicting breast cancer outcomes among patients in Tunisia was analyzed. It was found that a high Ki-67 index (>20%) was associated with younger age at diagnosis, lymph node involvement, and higher tumor grade, resulting in a higher risk of disease recurrence. For the luminal subtype, a 30% cut-off was used, which proved useful in predicting patient survival. The findings suggest that high Ki-67 levels may be a significant prognostic indicator in aggressive forms of breast cancer, helping to identify patients in need of more intensive oncological care [128]. Next, in research led by Erić et al., the focus was on analyzing breast cancer in young women (under 40 years old) compared to older patients (over 60 years old). It was found that the younger group had a higher Ki-67 index (averaging 25% compared to 10% in older patients), which correlated with more aggressive forms of the tumor. These results indicate that a higher Ki-67 index may signify a poorer prognosis and is characteristic of younger patients with more aggressive cancers [129]. Blessin et al. developed an automated system to assess the Ki-67 index using artificial intelligence and fluorescent immunohistochemistry, tested in a large cohort of prostate cancer patients. The results showed that the Ki-67 index provided strong, independent prognostic information, and a high Ki-67 level correlated with higher Gleason scores and aggressive disease progression. The study highlights that automated Ki-67 assessment could improve diagnostic reliability and precision, particularly in aggressive prostate cancers [130]. In turn, Taco Sanchez et al. evaluated the effectiveness of digital image analysis for assessing the Ki-67 index in breast cancer patients. The study compared visual assessment of Ki-67 with digital analysis, finding that digital analysis improved accuracy and reproducibility, especially in heterogeneous tumors. The results suggest that digital Ki-67 assessment may be a more reliable prognostic indicator, helping predict recurrence risk and tailoring treatment to the tumor characteristics in breast cancer patients [131]. In a study conducted by Mingzhu et al. in endometrial cancer, the combined ratio of estrogen and progesterone receptors to Ki-67 and p53 expression levels ([ER + PR]/[P53 + Ki-67]) was shown to outperform single markers in predicting disease recurrence. This combined approach allows for stratification of patients into distinct risk groups, with the ratio demonstrating a high level of accuracy in predicting three- and five-year recurrence-free survival rates. The integration of these biomarkers into clinical practice offers a robust tool for identifying patients at higher risk of recurrence, facilitating more personalized therapeutic strategies and intensive monitoring [132]. Similarly, studies in gastric cancer have highlighted the prognostic utility of combining Ki-67 with centromere protein H (CENP-H). Both biomarkers independently correlate with tumor aggressiveness, lymph node involvement, and advanced disease stages, but their combined evaluation provides a more reliable prognostic indicator. Patients with simultaneous overexpression of Ki-67 and CENP-H exhibit significantly poorer survival outcomes compared to those with lower levels of either marker. This synergistic effect underscores the importance of integrating multiple biomarkers to capture the complex biological interactions driving tumor progression [133].

Next, PD-L1 (Programmed Death-Ligand 1) is an immunosuppressive protein present on the surface of certain cancer cells, allowing tumors to evade detection by the immune system. PD-L1 expression is a significant prognostic factor in cancers such as lung cancer, melanoma, and renal cancer, where it indicates a higher risk of disease progression and poorer prognosis. A high level of PD-L1 also suggests the potential use of immunotherapy involving checkpoint inhibitors, such as PD-1/PD-L1 blocking antibodies, which opens new therapeutic possibilities for patients with advanced cancers [134,135].

**Figure 4:**
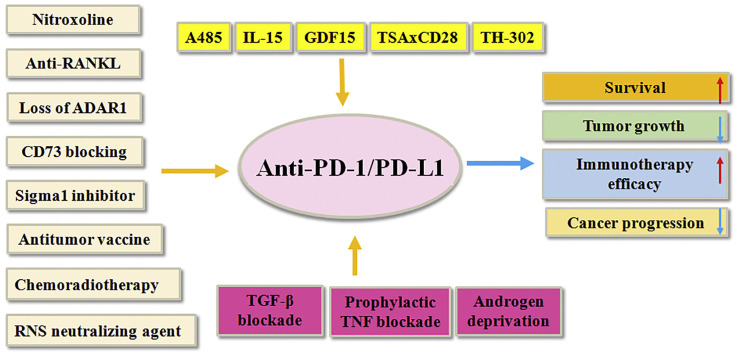
Anti-PD-1/PD-L1 in combination with other therapies [136].

Combining anti-PD1/PD-L1 antibodies with additional prostate cancer therapies can yield promising antitumor effects (Figure 4) [136].

A study conducted by Eichhorn et al. analyzed PD-L1 expression in patients with stage pN1 non-small cell lung cancer (NSCLC) who underwent resection surgery. The goal was to understand the impact of PD-L1 on survival after surgical treatment and adjuvant chemotherapy. It was shown that patients with low PD-L1 expression (>10%) had significantly better survival, especially those with adenocarcinoma, who showed a favorable response to adjuvant therapy. The findings suggest that PD-L1 testing may be useful in identifying patients with a better prognosis following surgical and adjuvant treatments, particularly in cases with adenocarcinoma histology [137]. Tuminello and team conducted a meta-analysis combining results from 40 studies evaluating the relationship between PD-L1 expression and survival in NSCLC patients after surgical resection. The analysis showed that high PD-L1 expression (regardless of cut-off values used) was associated with poorer overall survival (OS). The authors suggested that PD-L1 may serve a prognostic role in NSCLC and could be a potentially useful biomarker for predicting response to immunotherapy post-surgery. Furthermore, the results support further research into PD-L1 as a predictive biomarker for adjuvant immunosuppressive therapy [138]. Next, Chen et al. investigated the correlation between PD-L1 expression and clinical outcomes in breast cancer patients who received neoadjuvant chemotherapy (NACT). The analysis showed that high PD-L1 expression was associated with longer disease-free survival (DFS) and overall survival (OS) compared to patients with low PD-L1 expression. These results suggest that PD-L1 may act as a positive prognostic factor in breast cancer patients undergoing neoadjuvant therapy, which can impact clinical decisions and disease prognosis [139].

BCL-2 (B-cell lymphoma 2) is an anti-apoptotic protein that plays a crucial role in cancer prognosis, particularly due to its influence on cell survival pathways and its ability to inhibit programmed cell death. Overexpression of BCL-2 is often observed in various cancers, where it is associated with tumor resistance to apoptosis and an increased capacity for cell survival under stress conditions. In cancers such as breast, lung, and lymphoid malignancies, BCL-2 has demonstrated significant prognostic implications [140,141]. High levels of BCL-2 expression are frequently correlated with longer survival and a less aggressive tumor phenotype, especially in cases with positive hormone receptor status. However, the prognostic value of BCL-2 can vary across cancer types, as its expression may indicate resistance to certain chemotherapies, impacting treatment decisions. By serving as a potential marker of favorable prognosis, particularly in hormone-responsive cancers, BCL-2 enables more precise risk stratification and supports the development of personalized therapeutic approaches [142,143]. Moreover, the BCL-2 protein plays a critical role in inhibiting apoptosis, making it a significant therapeutic target in lymphoma treatment. Studies on the U-2946 cell line in DLBCL revealed that its unique profile (MCL1 overexpression and lack of BCL-2 expression) results in resistance to BCL-2 inhibitors such as ABT-263, highlighting the need for therapy personalization based on the molecular profile of the tumor [144]. In classical Hodgkin lymphoma (cHL), high BCL-2 expression has been identified as a negative prognostic factor. The use of venetoclax, a BCL-2 inhibitor, demonstrated effectiveness in cHL cell lines, especially in combination with drugs like vincristine or doxorubicin [145]. In mantle cell lymphoma (MCL), research on PRMT5 inhibition showed that activating pro-apoptotic BCL-2 family genes such as BAX enhances cell sensitivity to venetoclax. Combined therapy with PRMT5 inhibitors and venetoclax exhibited synergistic effects, improving survival in preclinical models [146].

**Figure 5:**
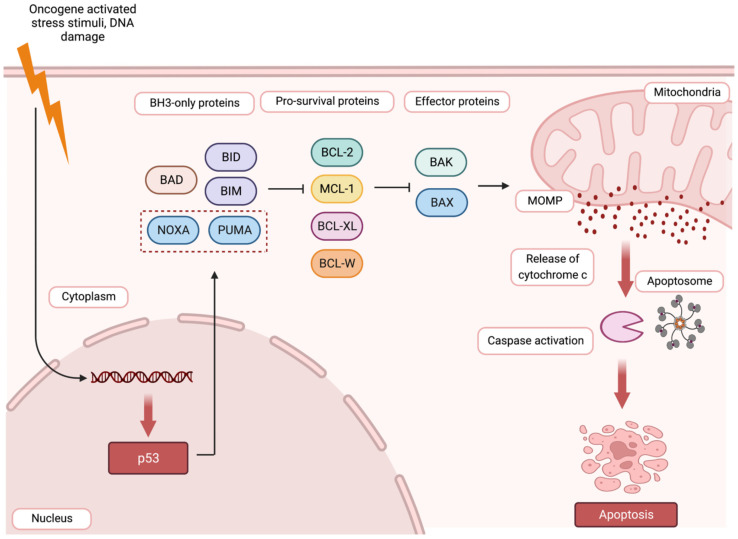
Intrinsic apoptosis pathway involving BCL-2 family proteins, where cellular stress triggers pro-apoptotic activation, cytochrome c release, and the caspase cascade, leading to cell death [140].

Figure 5 illustrates the intrinsic apoptosis pathway regulated by the BCL-2 protein family. This process is activated in response to cellular stress, such as DNA damage, leading to an increase in BH3-only proteins (e.g., PUMA, NOXA). These proteins bind and neutralize pro-survival BCL-2 proteins, releasing the pro-apoptotic proteins BAX and BAK. Once activated, BAX and BAK form pores in the mitochondrial membrane, allowing cytochrome c to be released, which activates the apoptosome and caspase cascade, resulting in controlled cell death [140].

Anagnostou et al. investigated BCL-2 expression in patients with non-small cell lung cancer (NSCLC) to assess its impact on survival outcomes. Analyzing two patient groups, they found that high BCL-2 expression was associated with longer survival, particularly in NSCLC cases with non-squamous histology. Patients with elevated BCL-2 levels demonstrated a better prognosis, suggesting BCL-2 as a potential prognostic factor in NSCLC, particularly useful for refining patient stratification [147]. Similarly, Dawson et al. conducted a large-scale study involving over 11,000 early-stage breast cancer patients to evaluate BCL-2 as an independent prognostic factor. Their findings indicated that high BCL-2 expression was linked to improved survival outcomes, regardless of estrogen receptor (ER) status or type of adjuvant therapy. This study underscores BCL-2 as a strong, independent indicator of favorable prognosis, with potential to enhance prognostic models in breast cancer [148]. Next, Al-Alem et al. assessed BCL-2 expression in patients with invasive breast cancer and examined its correlation with survival, hormone receptor status, and tumor grade. They found that BCL-2 expression was associated with longer survival and reduced tumor aggressiveness, particularly in patients with lower tumor grades and positive hormone receptor status. This study identifies BCL-2 as a favorable prognostic marker, highlighting its potential in improving patient stratification [149]. In the study by Bilalovic et al., the prognostic value of the BCL-2 protein in patients with invasive breast cancer was analyzed. Seventy-one cases were examined to assess BCL-2 expression and its association with other prognostic indicators, such as estrogen (ER) and progesterone (PR) receptors, tumor size, and lymph node status. The results showed that high BCL-2 expression was significantly associated with longer overall survival (OS) and relapse-free survival (RFS), independent of tumor size and lymph node status. Statistical analysis also revealed a positive correlation between BCL-2 expression and the presence of ER and PR receptors, suggesting that BCL-2 may be a marker of favorable prognosis, particularly in hormone-dependent types of breast cancer. These findings imply that BCL-2, although anti-apoptotic, may paradoxically reduce tumor aggressiveness, resulting in longer patient survival. According to the authors, this mechanism could be linked to BCL-2’s modulatory effects on cancer cell proliferation and the coexistence of other BCL-2 family proteins, such as Bax, which may counterbalance BCL-2’s effect [150].

### 3.2. Prognostic Proteins in Personalizing Therapy

The use of protein biomarkers in personalized cancer therapy represents a transformative approach, enabling treatments to be tailored specifically to the unique molecular profile of an individual’s tumor. Prognostic biomarkers, such as RAS mutation status or HER2 protein expression, provide insights into the risk of disease progression and are essential in selecting targeted therapies that deliver greater efficacy in certain cancer subtypes. For instance, in colorectal cancer, the presence of a RAS mutation signifies that therapies targeting the epidermal growth factor receptor (EGFR) are unlikely to be effective, allowing clinicians to avoid such treatments and instead focus on alternative therapeutic strategies that are better suited to the patient’s genetic profile. This targeted approach reduces unnecessary treatments, potential side effects, and allows for a more efficient therapeutic focus [151,152,153].

In breast and gastric cancers, overexpression of the HER2 protein enables the application of trastuzumab, a targeted therapy that binds specifically to the HER2 receptor on tumor cells. By blocking the signaling pathways that drive uncontrolled tumor growth, trastuzumab offers a highly effective treatment option for HER2-positive patients, often leading to improved outcomes compared to conventional therapies. HER2-positive patients can experience better control over tumor progression and longer survival times as a result of this targeted approach. The integration of these biomarkers into clinical practice not only enhances therapeutic precision but also optimizes the overall treatment process, helping to maximize efficacy, minimize unnecessary interventions, and improve quality of life for cancer patients [154,155].

Personalized cancer therapy, despite its advantages, encounters significant limitations. One of the main challenges is the development of resistance to targeted therapies, where cancer cells can develop mechanisms to evade drug action, leading to disease progression. Additionally, heterogeneity in biomarker expression within tumors can result in varied responses to therapy even in the same patient. This intra-tumoral diversity complicates treatment planning and underscores the need for more comprehensive diagnostic approaches to ensure the effectiveness of personalized interventions. Research indicates that heterogeneity in gene expression within tumors significantly impacts biomarker identification and therapy efficacy. Adaptive methods, such as aGRP, have demonstrated better performance in exploring this heterogeneity, which is crucial for capturing subtle yet consistent changes in gene expression in tumors [156]. Moreover, resistance to targeted therapies remains a major challenge in cancer treatment. Understanding the mechanisms of resistance, both intrinsic and extrinsic, can lead to the development of strategies that enhance therapy efficacy and improve patient outcomes [157]. Intra-tumoral heterogeneity, manifesting as differences in biomarker expression, can lead to inconsistencies in diagnostic results and impact clinical decisions. Studies on breast cancer have shown that such heterogeneity can be a source of discrepancies in biomarker status assessment, emphasizing the need for more precise diagnostic methods [158].

## 4. Proteins in Targeted Therapy in Oncology

Targeted therapy in oncology is based on a specific therapeutic intervention aimed at key proteins responsible for the development and progression of tumors. Unlike traditional methods such as chemotherapy or radiotherapy, which affect both cancerous and healthy cells, targeted therapy acts selectively, focusing on specific proteins or signaling pathways that are crucial to the functioning of cancer cells. The primary targets of targeted therapy include membrane receptors, tyrosine kinases, signaling proteins, and transcription factors that play essential roles in regulating the cell cycle, proliferation, and mechanisms of immune evasion. By precisely targeting these proteins, targeted therapy significantly increases treatment effectiveness while minimizing adverse effects. Targeted therapy, directed at specific molecular mechanisms responsible for disease progression, plays a pivotal role in modern oncology, yet its efficacy is limited by the development of drug resistance and the necessity of combination therapies. Drug resistance, both primary, resulting from the molecular heterogeneity of tumors, and secondary, acquired through selective pressure exerted by treatment, remains one of the major challenges in this therapeutic strategy [159]. These mechanisms include mutations in target genes, activation of alternative signaling pathways, and alterations in the tumor microenvironment [160]. In response, combination therapies are increasingly employed, allowing for simultaneous blockade of multiple signaling pathways, thereby reducing the likelihood of selecting resistant subclones. For instance, the combination of BRAF and MEK inhibitors in melanoma treatment significantly prolongs survival compared to monotherapy [161]. Despite these benefits, combination therapies come with additional challenges, such as toxicity, pharmacokinetic interactions, and increased treatment costs [162].

### 4.1. Mechanisms of Action of Targeted Therapies

Targeted therapies aim to disrupt specific signaling pathways or protein functions essential for tumor development. One of the primary mechanisms of action involves inhibiting receptors and tyrosine kinases, which play a crucial role in the proliferation and survival of cancer cells [163]. Drugs such as imatinib, trastuzumab, and gefitinib inhibit the activity of specific proteins or receptors, like BCR-ABL, HER2, and EGFR, responsible for the growth and division of cancer cells. This mechanism of action blocks these proteins’ functions, leading to the inhibition of signaling pathways and, ultimately, the cessation of cancer cell proliferation [164]. Another key mechanism is the use of monoclonal antibodies that bind to specific receptors on the surface of cancer cells, blocking growth signals and stimulating an immune response. Monoclonal antibodies, such as trastuzumab, bevacizumab, and rituximab, are widely used in treating various cancer types, including breast cancer, colorectal cancer, and lymphoma, demonstrating high efficacy due to their ability to specifically target essential proteins in cancer cells [165,166]. The mechanisms of action of targeted therapies in oncology are based on the specific influence on key proteins and signaling pathways that play critical roles in the growth, proliferation, and survival of cancer cells. These therapies focus on halting tumor growth, disrupting intercellular communication, and weakening the mechanisms by which tumors evade immune responses. Major mechanisms of targeted therapy include tyrosine kinase inhibition, membrane receptor inhibition, monoclonal antibody therapy, and interference with proteins regulating angiogenesis and immunotherapy. Each of these mechanisms targets specific proteins or cellular structures essential to the functioning of tumors [167,168].

### 4.2. Inhibition of Tyrosine Kinases

Tyrosine kinases are enzymes that play a key role in cellular signaling, regulating processes such as cell growth, differentiation, and survival. In cancer cells, these kinases are often mutated or overexpressed, leading to excessive activation of proliferative pathways and increased resistance to apoptosis [169]. Tyrosine kinase inhibitors (TKIs) are designed to bind to the active site of the kinase, blocking its ability to transmit growth signals. An example is imatinib, a BCR-ABL tyrosine kinase inhibitor used in the treatment of chronic myeloid leukemia (CML) [170,171]. In CML, chromosomal translocation creates the oncogenic BCR-ABL protein, a tyrosine kinase with uncontrolled activity. Imatinib binds to the active site of BCR-ABL, halting cancer cell proliferation and restoring control over the cell cycle [172]. Similarly, EGFR kinase inhibitors, such as gefitinib and erlotinib, inhibit the EGFR receptor, which is commonly present in cancers of the lung, colon, and pancreas [173].

**Figure 6:**
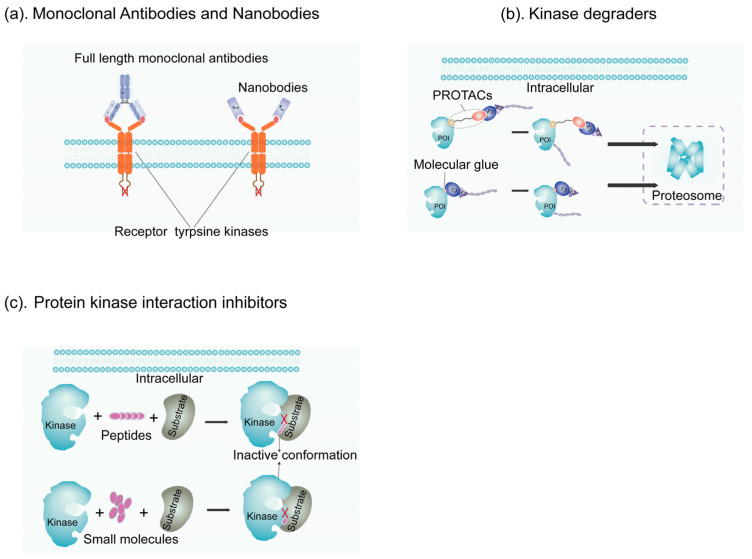
Mechanisms of kinase-targeted cancer therapies: (**a**) monoclonal antibodies and nanobodies, (**b**) kinase degraders like PROTACs and molecular glue, and (**c**) protein–kinase interaction inhibitors (PKIIs) [174].

Figure 6 illustrates various mechanisms of action for kinase-targeted cancer therapies, including monoclonal antibodies, nanobodies, kinase degraders, and protein–kinase interaction inhibitors. Monoclonal antibodies and nanobodies block cell signaling by binding to the extracellular domain of receptors; nanobodies, being single-domain antibodies (VHH), lack the Fc region. Kinase degraders, such as PROTACs, work by linking E3 ligases to target proteins (POI), inducing their degradation. Molecular glues are small molecules that bind to E3 ligase receptors and promote protein–protein interactions, leading to the degradation of target proteins. Protein–kinase interaction inhibitors (PKIIs), composed of small molecules or linear peptides, inhibit interactions between kinases and their substrates, making them ideal for disrupting protein–protein interactions [174].

The study by Zhou et al. examined the safety and efficacy of combining stereotactic radiotherapy (SRT) with third-generation tyrosine kinase inhibitors in advanced metastatic lung cancer with EGFR mutations. The results indicated that adding SRT to EGFR-TKI significantly extended progression-free survival (PFS) in patients with focal metastases, highlighting the effectiveness of combining EGFR-TKI with other targeted therapies to improve clinical outcomes [175]. Li et al. focused on resistance that emerges during EGFR-TKI treatment in non-small cell lung cancer (NSCLC) with EGFR mutations. The findings showed that BRAF gene mutations can lead to acquired resistance to EGFR-TKI, and combining RAF/MEK inhibitors with EGFR-TKI extends survival in patients with EGFR-TKI resistance. This strategy emphasizes the importance of a multifactorial therapeutic approach and suggests the potential of using triple-targeted therapies for resistant cancers [176]. The study by Toh et al. proposed a novel approach to increasing cancer cell sensitivity to EGFR inhibitors by using extracellular vesicles containing an isoform variant of EGFR (IsoD). This EGFR variant enhances sensitivity to EGFR-TKI treatment through synergy in cellular endosomes. This mechanism presents a promising tool to support the action of tyrosine kinase inhibitors and may offer clinical benefits in various types of TKI-resistant cancers [177].

### 4.3. Membrane Receptor Inhibition

Membrane receptor inhibition is a targeted approach in cancer therapy that focuses on blocking specific receptors, such as HER2 and EGFR, which are involved in cellular growth and differentiation signaling. In many cancers, these receptors are overexpressed or mutated, leading to continuous activation of signaling pathways that drive uncontrolled cell proliferation and survival. By targeting these receptors, therapies can interrupt this excessive signaling and reduce tumor growth [178]. Monoclonal antibodies are the most commonly used agents in membrane receptor inhibition. They are designed to specifically bind to the extracellular domain of cancer cell receptors, preventing the natural growth factors from binding and stopping receptor dimerization—a necessary step for activating downstream signals [179]. This blockade effectively shuts down the aberrant signaling that contributes to cancer progression. In some cases, this inhibition not only stops cell division but also triggers cell death, directly affecting the tumor’s ability to grow and spread. Membrane receptor inhibition with monoclonal antibodies has become a cornerstone in targeted cancer therapy, offering a precise mechanism to neutralize the specific drivers of tumor growth at the cell surface level [180,181].

Nasarre et al. studied the effect of inhibiting Neuropilin-1 (NRP1) on glioma growth using a synthetic peptide that disrupts the transmembrane domain of NRP1. Their findings showed that blocking NRP1 reduced angiogenesis and cell migration, ultimately inhibiting tumor growth [182]. Similarly, Roth and his team focused on NRP1 dimers, demonstrating that disrupting the dimerization motif of NRP1 weakened VEGF and Sema3A signaling, which are essential for glioma cell migration and invasion [182]. In a study on Plexin-A1, the authors examined how blocking this receptor affected semaphorin pathways in brain tumors. The results indicated that Plexin-A1 inhibition reduced glioma invasiveness, suggesting that this receptor may be an effective target for limiting tumor spread [183]. Meanwhile, Arpel and colleagues analyzed NRP1 inhibition in breast cancer models using a peptide that restricted NRP1 signaling, leading to a reduction in tumor growth and metastasis. These findings suggest that inhibiting membrane receptors like NRP1 and Plexin-A1 can effectively hinder key processes associated with cancer cell proliferation and migration, making these receptors promising therapeutic targets in oncology [184].

### 4.4. Kinase Degraders and Molecular Glues

In recent years, the development of targeted therapies in oncology has significantly accelerated with the introduction of innovative strategies such as kinase degraders and molecular glues. These approaches represent a major breakthrough in cancer treatment, offering new possibilities for overcoming therapy resistance and targeting proteins previously considered difficult to address [185]. Kinase degraders are small molecules designed for the selective elimination of protein kinases through a ubiquitin–proteasome-dependent degradation mechanism. Unlike traditional kinase inhibitors, which merely block enzymatic activity, degraders lead to the complete removal of the target protein from the cell. This mechanism involves the degrader binding to both the kinase and an E3 ligase, facilitating protein ubiquitination and subsequent degradation in the proteasome [186]. An example of this technology is PROTACs (Proteolysis Targeting Chimeras), which consist of two ligands linked by a linker: one ligand binds the kinase, while the other recruits the E3 ligase. By doing so, kinase degraders can effectively eliminate oncogenic kinases, opening new avenues for cancer treatment, especially in cases of resistance to inhibitors [187,188].

Molecular glues are another promising approach in targeted therapy. These are small molecules that stabilize or induce interactions between proteins, leading to their functional modification or degradation. In the context of cancer therapy, molecular glues can promote interactions between E3 ligases and target proteins, resulting in their ubiquitination and degradation. Unlike PROTACs, which require two separate ligands, molecular glues act as single molecules that modify protein surfaces, facilitating their interactions. A notable example is lenalidomide, which binds the E3 ligase cereblon (CRBN) and promotes the degradation of proteins such as Ikaros and Aiolos, a mechanism utilized in the treatment of multiple myeloma [189,190]. Both approaches—kinase degraders and molecular glues—offer innovative therapeutic opportunities, particularly in cases where traditional inhibitors prove ineffective due to mutations or resistance. Kinase degraders enable the complete elimination of oncogenic kinases, while molecular glues can target difficult-to-address proteins. These advancements mark a significant step forward in the development of more precise and effective cancer therapies [191,192].

### 4.5. Use of Monoclonal Antibodies

Monoclonal antibodies are specialized immunoglobulins engineered to selectively bind to specific proteins or receptors on the surface of cancer cells, allowing for targeted intervention in tumor growth and survival. These antibodies operate through multiple mechanisms to combat cancer more effectively. One primary mode of action is blocking receptors on cancer cells that transmit signals for proliferation and survival; by inhibiting these pathways, monoclonal antibodies can effectively halt tumor growth. Additionally, monoclonal antibodies can “coat” or opsonize cancer cells, marking them for destruction and enhancing their recognition and elimination by the immune system through processes such as antibody-dependent cellular cytotoxicity (ADCC) [193,194]. Moreover, some monoclonal antibodies are designed as drug conjugates (antibody–drug conjugates or ADCs) that deliver cytotoxic agents or radioactive molecules directly to cancer cells, providing a highly targeted approach that minimizes damage to surrounding healthy tissue. This targeting not only increases the efficacy of the therapeutic agent but also reduces side effects compared to conventional therapies. Overall, monoclonal antibodies offer a precise and multi-faceted approach to cancer treatment, contributing to the personalization of therapy and improving patient outcomes [195,196].

Trastuzumab, also known as Herceptin, is a monoclonal antibody used in targeted therapy for cancers with HER2 overexpression, especially breast cancer. Studies have demonstrated its effectiveness in inhibiting cancer cell proliferation by specifically binding to HER2 receptors, leading to blocked growth signaling and induction of apoptosis in cancer cells [197]. The study by Chang et al. focused on treating HER2-positive metastatic colorectal cancer using a combination of trastuzumab and pyrotinib. This combination was shown to extend patient survival and demonstrate significant antitumor effects, especially in patients with RAS wild-type cancer. In the patient group, an objective response rate of 50% was achieved along with long-term therapeutic benefits, highlighting trastuzumab’s value as a key component in treating HER2-positive metastases [198]. Further research by Kobzeva and colleagues designed an ADC conjugate based on trastuzumab using a self-destructive disulfide linker, enabling controlled drug release within cancer cells and reducing systemic toxicity. In vivo tests confirmed the conjugate’s higher efficacy on HER2-positive cell lines and mouse models compared to trastuzumab alone [199]. Overall, research on trastuzumab underscores its versatility as a monoclonal antibody for treating HER2-positive cancers. Both combinations with other kinase inhibitors and innovative drug conjugates increase its effectiveness, opening possibilities for better personalization in cancer therapy [200,201].

Bevacizumab is a monoclonal antibody that specifically targets vascular endothelial growth factor A (VEGF-A), a protein involved in angiogenesis—the formation of new blood vessels that tumors need to grow [202]. By binding to VEGF-A, Bevacizumab inhibits the VEGF receptor signaling pathway, reducing blood supply to the tumor, which can slow its growth and spread [203]. This approach has been essential in treating metastatic cancers, particularly colorectal cancer (CRC) and non-small cell lung cancer (NSCLC) [204].

Research conducted by Li et al. demonstrated that, while Bevacizumab significantly improves outcomes in metastatic CRC by reducing blood vessel formation, its efficacy can be compromised by resistance mechanisms. Their study highlights that tumor-derived lactate may contribute to Bevacizumab resistance by enhancing autophagy—a cellular survival mechanism. Elevated lactylation levels were associated with poorer survival in patients with CRC treated with Bevacizumab, suggesting a need for combination therapies to counteract resistance and improve patient outcomes [205]. Socinski et al. studied the combination of Bevacizumab with other therapeutic agents, such as Atezolizumab (an anti-PD-L1 antibody), in patients with NSCLC. This study observed that adding Bevacizumab to a regimen including chemotherapy and immunotherapy (Atezolizumab) led to a notable improvement in progression-free survival and overall survival, particularly in patients with high PD-L1 expression. This combination exploits both angiogenesis inhibition and immune modulation, offering a robust strategy for treating advanced NSCLC [206]. Another study found that Bevacizumab’s effectiveness could be enhanced when combined with treatments that inhibit histone lactylation, as lactylation was shown to promote CRC cell survival and resistance under hypoxic conditions induced by antiangiogenic therapy. By inhibiting histone lactylation, researchers observed a significant increase in the sensitivity of CRC cells to Bevacizumab, suggesting that integrating metabolic and epigenetic targeting may counteract resistance to Bevacizumab-based therapies [207].

### 4.6. Interference with Angiogenesis Signaling Pathways

Targeting angiogenesis as a therapeutic strategy in cancer treatment involves blocking angiogenic processes to cut off nutrient and oxygen supply to the tumor, slowing its growth and limiting its ability to metastasize. Angiogenesis is a regulated biological process that supports the formation of new blood vessels, controlled by a balance between pro-angiogenic factors, such as VEGF (vascular endothelial growth factor), PDGF (platelet-derived growth factor), and angiopoietins, and anti-angiogenic factors. In cancers, this balance shifts towards excessive production of pro-angiogenic factors, which fuel intense cancer cell proliferation and enhance metastatic potential [208]. Anti-angiogenic therapies focus on disrupting the signaling pathways associated with angiogenesis by blocking critical proteins and receptors involved in this process. A key role is played by VEGF inhibition, but modern targeted therapies also cover a broader range of factors that participate in angiogenesis [209]. For example, blocking VEGFR and PDGFR receptors leads to destabilization of the tumor’s vascular network, which restricts blood flow and results in tumor hypoxia. Other targets include integrin receptors, which are involved in endothelial cell adhesion to the extracellular matrix, impacting vascular structural integrity and reducing nutrient accessibility for the tumor. Significantly, newly formed vessels within the tumor are often unstable, distorted, and highly permeable, creating a hypoxic microenvironment. Although hypoxia restricts tumor growth, it may also trigger adaptive mechanisms that paradoxically increase tumor aggressiveness and metastatic potential [210,211]. Therefore, research on anti-angiogenic therapies increasingly explores combination strategies, pairing anti-angiogenic drugs with immunotherapy, chemotherapy, or radiotherapy. These combinations can act synergistically, simultaneously reducing the risk of resistance to anti-angiogenic therapy and enabling a more comprehensive approach to fighting cancer [212]. Further research focuses on optimizing the timing and dosing of anti-angiogenic drugs to maximize therapeutic efficacy and minimize side effects. For example, studies on pulsatile therapy, which involves periodically blocking angiogenesis, suggest benefits in reducing tumor adaptation and toxicity to healthy tissues. Periodic angiogenesis inhibition allows for the regeneration of normal blood vessels, which may also benefit the patient by reducing side effects and improving quality of life. Challenges in anti-angiogenic therapy also include the development of biomarkers to predict treatment response and monitor therapeutic progress. Biomarkers can provide valuable information about the effectiveness of angiogenesis inhibition, helping to better tailor treatment to the individual needs of the patient. In this way, personalizing anti-angiogenic therapies is becoming increasingly achievable, potentially improving treatment outcomes and minimizing unwanted effects across a wide range of cancers [213,214].

**Figure 7:**
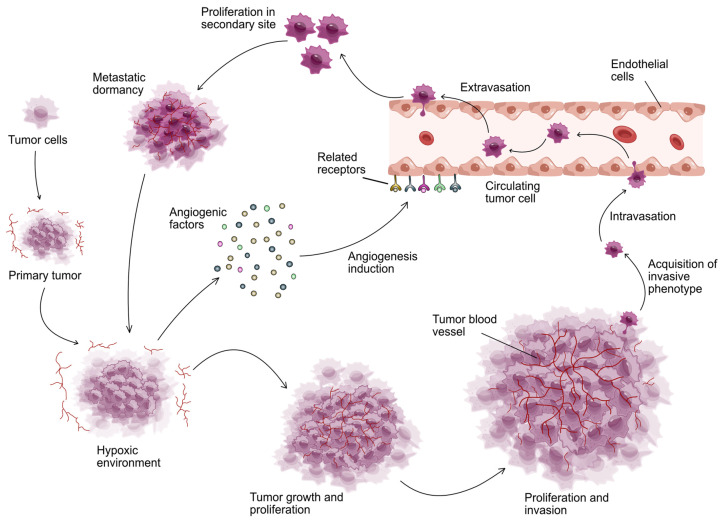
Scheme of cancer progression driven by hypoxia-induced angiogenesis, enabling tumor growth, survival, and metastasis through the formation of new blood vessels [215].

Figure 7 depicts how angiogenesis contributes to cancer progression. As the tumor rapidly expands, it depletes its oxygen supply, creating a hypoxic microenvironment. This low-oxygen condition triggers the release of pro-angiogenic factors such as VEGF, PDGF, FGF, and angiopoietin, which promote the formation of new blood vessels. These new vessels supply the tumor with essential oxygen and nutrients, allowing it to grow and survive. As the tumor evolves to a more aggressive state, it not only continues to grow and stimulate angiogenesis but also spreads by invading nearby tissues and metastasizing to distant sites via the bloodstream [215]. Moreover, the combination of antiangiogenic therapies with immunotherapy or chemotherapy is gaining increasing interest in oncology, as it integrates different mechanisms of action, enhancing treatment efficacy and reducing resistance. Antiangiogenic therapies, aimed at inhibiting tumor neovascularization by blocking signaling pathways such as VEGF, limit the supply of nutrients and oxygen to cancer cells, thereby reducing tumor growth [216]. However, their efficacy is often constrained by the development of adaptive resistance mechanisms, such as the recruitment of alternative pathways or changes in the tumor microenvironment [217]. Combining antiangiogenic therapy with immunotherapy can overcome these limitations by affecting the immune system. Antiangiogenic therapies normalize tumor blood vessels, improving drug delivery and enabling better access of immune cells to the tumor tissue [218]. At the same time, reducing immunosuppression in the tumor microenvironment, achieved through VEGF inhibition, enhances the effectiveness of immune checkpoint inhibitors such as anti-PD-1/PD-L1 and anti-CTLA-4 [219]. For example, the combination of a VEGF inhibitor (bevacizumab) with immunotherapy and chemotherapy significantly extended overall survival in lung cancer patients compared to chemotherapy alone [220]. Similarly, the combination of antiangiogenic therapies with chemotherapy enables synergistic effects. Antiangiogenic drugs increase vascular permeability within the tumor, improving the delivery of cytotoxic agents to cancer cells. The combination of bevacizumab with fluoropyrimidine-based chemotherapy in the treatment of colorectal cancer resulted in improved outcomes, both in progression-free survival and overall survival [221].

### 4.7. Delivery of Toxins with the Assistance of Antibodies—Immunotoxins

Immunotoxins represent a targeted cancer therapy approach that combines the specificity of antibodies with the potency of cytotoxic agents. In this strategy, antibodies are conjugated with toxins to selectively deliver these lethal agents directly to cancer cells while minimizing effects on healthy tissue. The antibodies used in immunotoxins are engineered to bind specifically to antigens overexpressed on the surface of tumor cells, which enables the precise targeting of cancerous tissue [222,223]. Once the immunotoxin binds to its target receptor on a cancer cell, it is internalized, releasing the toxin within the cell. The toxin then disrupts critical cellular functions, such as protein synthesis, ultimately leading to cell death. A key advantage of immunotoxins is their ability to selectively attack tumor cells, reducing the systemic toxicity often seen with traditional chemotherapeutics. Immunotoxins employ a range of cytotoxic agents, including bacterial or plant-derived toxins like Pseudomonas exotoxin or ricin, which are modified to ensure targeted delivery and safety [224]. This approach is especially promising for cancers that overexpress specific receptors, such as HER2, EGFR, or EphA2, allowing immunotoxins to deliver highly potent agents selectively. Recent developments in the design and engineering of immunotoxins have shown significant potential in improving therapeutic efficacy, making this strategy a valuable avenue in personalized cancer treatment [225,226].

Xi et al. developed an immunotoxin targeting TROP-2, a protein overexpressed in various epithelial tumors. The study utilized a shark-derived VNAR antibody, a small and highly stable antigen receptor that selectively binds to TROP-2. By fusing VNAR-5G8 to a modified version of Pseudomonas exotoxin (PE38), they created an immunotoxin that effectively binds and internalizes into TROP-2-expressing cells. In vitro and in vivo testing demonstrated that this immunotoxin induced significant apoptosis in breast cancer cells with minimal toxicity to non-cancerous cells. This study highlights the potential of shark-derived antibodies for improved tumor penetration and specificity in immunotoxin-based therapies [227]. Barazesh et al. explored the design and cytotoxic efficacy of a humanized immunotoxin (GnRH-DFF40) targeting cells expressing the GnRH receptor, often overexpressed in breast cancer cells. This fusion protein combines GnRH for cell-specific targeting with DFF40, a DNA fragmentation enzyme. Laboratory experiments showed dose-dependent cytotoxicity across three breast cancer cell lines, demonstrating apoptosis induction and migration inhibition in cancer cells. The study underscores the viability of GnRH-DFF40 for targeted breast cancer treatment, presenting a promising candidate for further clinical investigation due to its selective toxicity towards GnRH receptor-positive cancer cells [228]. Another study by Faraz et al. investigated the use of immunotoxins targeting the receptor tyrosine kinase EphA2, which is overexpressed in many cancers, including breast cancer. The researchers developed two versions of an immunotoxin combining an antibody fragment against EphA2 with a plant-derived toxin, ricin A subunit, creating EphA2-C-Ricin (C-terminal orientation) and EphA2-N-Ricin (N-terminal orientation). These immunotoxins were tested in vitro on three cell lines: MDA-MB-231 (high EphA2 expression), MCF-7 (low EphA2 expression), and HEK293 (very low EphA2 expression). Results indicated that EphA2-C-Ricin showed superior binding, internalization, and induction of apoptosis, particularly in MDA-MB-231 cells, as confirmed by Annexin V-Propidium iodide assays. This study underscores the potential of EphA2-targeted immunotoxins in selective cancer treatment, with the orientation of the antibody–toxin linkage playing a critical role in therapeutic efficacy [229]. In the study by Jang et al., a novel recombinant immunotoxin, HER2(scFv)-CRT, was developed to target HER2-positive cancer cells, specifically breast cancer. This immunotoxin combines crotamine, a toxin from rattlesnake venom with cytotoxic properties, with a single-chain variable fragment (scFv) derived from trastuzumab, a monoclonal antibody against HER2. Crotamine was linked to scFv to enhance the selectivity of the toxin specifically toward HER2-overexpressing cells. The immunotoxin, expressed and purified from Escherichia coli, was tested in vitro on HER2-positive breast cancer cell lines, showing increased cytotoxicity and specificity towards HER2-expressing cells compared to other cell lines. This approach exemplifies how combining targeted antibodies with potent toxins can selectively attack cancer cells while minimizing damage to healthy tissue [230]. In another study, Cerise et al. focused on developing anti-Mesothelin (MSLN) recombinant immunotoxins (RITs) for colorectal cancer (CRC), as well as other cancers expressing MSLN, such as pancreatic, ovarian, and mesothelioma tumors. The MSLN-targeted immunotoxin was created by fusing an anti-MSLN antibody fragment with Pseudomonas exotoxin A’s catalytic domain. Tests showed that CRC cell lines expressing MSLN responded effectively to this RIT, exhibiting cytotoxic effects comparable to those seen in pancreatic cancer studies. In a mouse model with SW48 CRC tumors, the MSLN-targeted RIT significantly reduced tumor volume. Although combining this RIT with the chemotherapeutic oxaliplatin did not enhance tumor regression, co-administration with actinomycin D led to a 90% reduction in tumor size and achieved full regression in 50% of cases. These findings suggest that MSLN-targeted immunotoxins, especially in combination with specific chemotherapeutics, could be a powerful option for treating CRC and other MSLN-expressing cancers [231].

Targeted therapy in oncology offers new treatment options by targeting specific proteins and signaling pathways essential for tumor growth. Mechanisms of action include inhibition of tyrosine kinases, blockade of membrane receptors, use of monoclonal antibodies, inhibition of angiogenesis, checkpoint immunotherapy and delivery of toxins via immunotoxins. Each of these mechanisms acts on specific targets, allowing precise therapeutic intervention, minimizing side effects and improving treatment efficacy. A summary of selected mechanisms is presented in Table 2.

## 5. Summary and Future Directions

Despite significant advancements in understanding and targeting proteins in oncology, key challenges remain in fully realizing the potential of protein-based diagnostics, prognostic markers, and targeted therapies. One of the most pressing challenges is the development of therapeutic resistance, where tumors adapt through alternative signaling pathways or additional mutations, reducing the efficacy of treatments targeting specific proteins. For instance, secondary mutations in tyrosine kinases or compensatory activation of parallel pathways can limit the long-term success of therapies like kinase inhibitors or monoclonal antibodies [232]. To address this, the development of combination therapies targeting multiple proteins or pathways simultaneously is critical, as these approaches may overcome tumor adaptations and enhance therapeutic efficacy [159]. In diagnostics and prognostics, tumor heterogeneity poses another significant challenge, with variability in protein expression across patients and within tumors impacting the reliability of protein-based biomarkers [233]. Advances in precision medicine, particularly through comprehensive genetic and proteomic profiling, are vital to address this heterogeneity. However, additional research is needed to refine these techniques, enabling more accurate predictions of patient responses to protein-targeted therapies. Emerging approaches, such as single-cell proteomics, offer exciting possibilities for dissecting tumor heterogeneity and identifying actionable biomarkers [234]. Future research in protein-targeted oncology is poised to focus on innovative combination strategies that integrate protein-based therapies with immunotherapy, chemotherapy, or radiation to enhance efficacy while reducing resistance [219]. Cutting-edge developments in bispecific antibodies and antibody–drug conjugates (ADCs) are particularly promising, offering the potential to simultaneously target multiple protein markers and deliver cytotoxic agents with high selectivity, thereby minimizing off-target effects [235]. Additionally, the integration of artificial intelligence (AI) and machine learning (ML) into oncology research represents a transformative avenue. These technologies can accelerate the discovery of novel biomarkers, predict patient responses, and optimize therapeutic regimens, paving the way for adaptive and highly personalized treatment strategies [236].

## Figures and Tables

**Table 1:** Comparison of Advantages and Limitations of Protein Biomarkers [82,83,84,85,86,87,88,89,90,91,92,93,94,95,96,97,98,99,100,101,102,103,104].

Criterion	Advantages	Limitations
Early detection	Allows for cancer detection at an early stage before clinical symptoms appear	Some biomarkers may not be present in early stages, leading to potential false-negative results
Monitoring therapy	Assists in assessing treatment effectiveness and detecting recurrences	Variability in biomarker levels among patients may complicate assessment of treatment response
Minimally invasive	Enables biomarker analysis in bodily fluids (e.g., blood, urine)	Biomarkers can sometimes be present in low concentrations in fluids, requiring highly sensitive assays
Specificity	Certain biomarkers are specific to particular cancers (e.g., HER2 in breast cancer)	Low specificity of some markers (e.g., PSA) can lead to false-positive results
Personalized therapy	Helps in tailoring targeted therapies based on specific biomarkers (e.g., HER2, PD-L1)	Limited access to certain biomarkers (costs and technology) restricts personalization in some cases
Cost and accessibility	Widely available biomarker tests in many oncology centers	High costs and limited availability of certain advanced tests
Standardization of methods	Standardization allows for result comparability across different laboratories	Lack of standardization in some cases results in discrepancies in results between facilities

**Table 2:** Summary of targeted therapy mechanisms, applications, and key considerations in oncology.

Mechanism	Description	Example Drugs	Primary Cancer Types	Advantages	Limitations
Inhibition of Tyrosine Kinases	Blocks tyrosine kinases involved in growth and survival pathways, stopping tumor cell proliferation.	Imatinib, Gefitinib, Erlotinib	CML, lung cancer, colorectal cancer	High specificity; effective in mutation-driven cancers	Resistance can develop; limited to kinase mutations
Blocking Membrane Receptors	Prevents growth signals by targeting overexpressed receptors on cancer cells.	Trastuzumab, Cetuximab	Breast cancer, head and neck cancer, colorectal cancer	Effective in receptor-driven cancers; enhances immune response	Requires receptor overexpression; limited to receptor-specific cancers
Monoclonal Antibodies	Targets specific proteins on cancer cells, triggering immune response or blocking growth.	Rituximab, Bevacizumab, Cetuximab	Lymphoma, colorectal cancer, lung cancer	Can deliver toxins selectively; immune-mediated action	High cost; potential for immune-related side effects
Angiogenesis Inhibition	Blocks vascular growth factor (VEGF) to starve tumors of nutrients and oxygen.	Bevacizumab, Sunitinib	Renal, lung, and colorectal cancers	Limits tumor growth by cutting off blood supply	Limited effect if tumor adapts; potential for resistance
Checkpoint Inhibition (Immunotherapy)	Reactivates T-cells to recognize and attack cancer cells by blocking immune checkpoints (e.g., PD-1/PD-L1).	Pembrolizumab, Nivolumab, Atezolizumab	Melanoma, lung cancer, kidney cancer	Long-lasting responses; high efficacy in immune-reactive tumors	High cost; immune-related adverse effects
Immunotoxins (Toxin Delivery)	Antibodies deliver toxins directly to cancer cells, leading to targeted cell death.	Brentuximab vedotin	Hodgkin lymphoma, leukemia	High specificity; limited impact on healthy cells	Can be limited to blood cancers; potential toxicity

## Data Availability

Not applicable.
